# The Philosophy of Health; or, an Exposition of the Physical and Mental Constitution of Man, &c.

**Published:** 1835-04-01

**Authors:** 


### The Philosophy of Health; or an Exposition of the Physical and Mental Constitution of Man, with a View to the Promotion of Human Longevity and Happiness. By *Southwood Smith*, M.D. Vol. I. pp. 408. 1835.

This is really a very clever work, and much do we regret that only the first volume is published; for we suspect that the second volume, treating on *mind*, will be still more interesting than this, whose object is *matter*. This work will be popular in one sense—but not in the ordinary sense. It will be much read, and much relished, by the comparatively few who read and *think*—but not by the multitude who read without reflection. Although there is not, perhaps, much in the volume which might claim the character of being perfectly new or original, yet the subjects are very often expressed in such an able and agreeable manner, that they strike us with an air of novelty, and seldom fail to rivet our attention. The second chapter, for example, in which the two lives, the animal and organic, are described and compared, may be read with great advantage by the medical student—aye, and the practitioner, too—as conveying the knowledge of a very curious and important subject in luminous language, and comprehensible by the meanest capacity. Not one-half of our medical brethren have a clear conception of the separate existence, yet intimate connexion, of the organic and animal lives—and a still smaller proportion have any idea of the part which this distinction and intimacy play in the animal economy, both in health and in disease. To them, therefore, we recommend a perusal of Dr. Smith's book —especially the second, third, and fourth chapters. The following extract from the second chapter, exhibiting a sketch of man's declension from the meridian of his mental and physical zenith, down to the setting of his sun in the ocean of oblivion, will convey a good idea of Dr. Smith's manner of handling his subjects.

"In man, the process of death is seldom altogether natural. It is generally rendered premature by the operation of circumstances which destroy life otherwise than by that progressive and slow decay which is the inevitable result of the action of organized structure. Death, when natural, is the last event of an extended series, of which the first that is appreciable is a change in the animal life and in the noblest portion of that life. The higher faculties fail in the reverse order of their development; the retrogression is the inverse of the progression, and the noblest creature, in returning to the state of non-existence, retraces step by step each successive stage by which it reached the summit of life.

In the advancing series, the animal is superadded to the organic life; sensation, the lowest faculty of the animal life, precedes ratiocination, the highest. The senses called into play at the moment of birth soon acquire the utmost perfection of which they are capable; but the intellectual faculties, later developed, are still later perfected, and the highest the latest.

In the descending series, the animal life fails before the organic, and its nobler powers decay sooner and more rapidly than the subordinate. First of all, the impressions which the organs of sense convey to the brain become less numerous and distinct, and consequently the material on which the mind operates is less abundant and perfect; but at the same time, the power of working vigorously with the material it possesses more than proportionally diminishes. Memory fails; analogous phenomena are less readily and less completely recalled by the presence of those which should suggest the entire train; the connecting links are dimly seen or wholly lost; the train itself is less vivid and less coherent; train succeeds train with preternatural slowness, and the consequence of these growing imperfections is that, at last, induction becomes unsound just as it was in early youth; and for the same reason, namely, because there is not in the mental view an adequate range of individual phenomena; the only difference being that the range comprehended in the view of the old man is too narrow, because that which he had learnt he has forgotten; while in the youth it is too narrow, because that which it is necessary to learn has not been acquired.

And with the diminution of intellectual power the senses continue progressively to fail: the eye grows more dim, the ear more dull, the sense of smell less delicate, the sense of touch less acute, while the sense of taste, immediately subservient to the organic function of nutrition, is the last to diminish in intensity and correctness, and wholly fails but with the extinction of the life it serves.

But the senses are not the only servants of the brain; the voluntary muscles are so equally; but these ministers to the master-power, no longer kept in active service, the former no longer employed to convey new, varied, and vivid impressions, the latter no longer employed to execute the commands of new, varied, and intense desires, become successively feebler, slower, and more uncertain in their action. The hand trembles, the step totters, and every movement is tardy and unsteady. And thus, by the loss of one intellectual faculty after another, by the obliteration of sense after sense, by the progressive failure of the power of voluntary motion; in a word, by the declining energy and the ultimate extinction of the animal life, man, from the state of maturity, passes a second time through the stage of childhood back to that of infancy; lapses even into the condition of the embryo: what the fœtus was, the man of extreme old age is: when he began to exist, he possessed only organic life; and before he is ripe for the tomb, he returns to the condition of the plant.

And even this merely organic existence cannot be long maintained. Slow may be the waste of the organic organs; but they do waste, and that waste is not repaired, and consequently their functions languish, and no amount of stimulus is capable of invigorating their failing action. The arteries are rigid and cannot nourish; the veins are relaxed and cannot carry on the mass of blood that oppresses them; the lungs, partly choked up by the deposition of adventitious matter, and partly incapable of expanding and collapsing by reason of the feeble action of the respiratory apparatus, imperfectly aërate the small quantity of blood that flows through them; the heart, deprived of its wonted nutriment and stimulus, is unable to contract with the energy requisite to propel the vital current; the various organs, no longer supplied with the quantity and quality of material necessary for carrying on their respective processes, cease to act; the machinery stops, and this is death.

And now, the processes of life at an end, the body falls within the dominion of the powers which preside universally over matter; the tie that linked all its parts together, holding them in union and keeping them in action, in direct opposition to those powers dissolved, it feels and obeys the new attractions to which it has become subject; particle after particle that stood in beautiful order fall from their place; the wonderful structures they composed melt away; the very substances of which those structures were built up are resolved into their primitive elements; these elements, set at liberty, enter into new combinations, and become constituent parts of new beings; those new beings in their turn perish; from their death springs life, and so the changes go on in an everlasting circle." 72.

The third chapter, however, is our favourite. In this, our author examines the ultimate object of organization and life—and with an amiable, but, we fear, an enthusiastic *optimism*, comes to the conclusion that this ultimate object is—PLEASURE.

"Two functions, sensation and voluntary motion, are combined in the animal life. Of these two functions, the latter is subservient to the former: voluntary motion is the servant of sensation, and exists only to obey its commands.

Is sensation, then, the ultimate object of organization? Simple sensation cannot be an ultimate object, because it is invariably attended with an ultimate result; for sensation is either pleasurable or painful. Every sensation terminates in a pleasure or a pain. Pleasure or pain, the last event in the series, must then be the final end.

Is the production of pain the ultimate object of organization? That cannot be, for the production of pain is the indirect, not the direct—the extraordinary, not the ordinary, result of the actions of life. It follows that pleasure must be the ultimate object, for there is no other of which it is possible to conceive. The end of organic existence is animal existence; the end of animal existence is sentient existence; the end of sentient existence is pleasurable existence; the end of life, therefore, is enjoyment. Life commences with the organic processes; to the organic are superadded the animal; the animal processes terminate in sensation; sensation ends in enjoyment; it follows, that enjoyment is the final end. For this every organ is constructed; to this every action of every organ is subservient; in this every action ultimately terminates.

And without a single exception in the entire range of the sentient creation, the higher the organized structure the greater the enjoyment, mediately or immediately, to which it is subservient. From its most simple to its most complex state, every successive addition to structure, by which function is rendered more elevated and perfect, proportionally increases the exquisiteness of the pleasure to which the function ministers, and in which it terminates.

Pleasure is the result of the action of living organs, whether organic or animal; pleasure is the direct, the ordinary, and the gratuitous result of the action of both sets of organs; the pleasure resulting from the action of the organs is conducive to their complete development, and thereby to the increase of their capacity for affording enjoyment; the pleasure resulting from the action of the organs, and conducive to their development, is equally conducive to the perpetuation of their action, and consequently to the maintenance of life; it follows, not only that enjoyment is the end of life, but that it is the means by which life is prolonged." 75.

Our author happily illustrates the connexion of the animal with the organic life, through the medium of the great sympathetic, and shews that, although the organs supplied by the ganglionic nerves, perform their functions without our knowledge, and without our consent; yet their healthy action communicates a pleasurable sensation to the whole of the animal life—and why? "Because the sentient mingle with organic nerves—because the sentient nerves are impressed by the actions of the organic organs. And how impressed? As long as the actions of the organic organs are sound, that is, as long as their processes are duly performed, the impression communicated to the sentient nerves is, in its nature, agreeable;—is, in fact, the PLEASURABLE CONSCIOUSNESS WHICH CONSTITUTES HEALTH." 81.

As, in this volume, Dr. S. does not enter at all into the converse of the picture—into the sufferings produced by the morbid workings of the ganglionic viscera, all appears in rosy tints, and the life of man is portrayed as an almost uninterrupted series of pleasurable sensations. Such would probably be the case, were we in a state of Nature—or at least in that state which the beneficent Author of our Being designed for us. But whether, in consequence of the original fall of man from a state of innocence, or from the vitiated condition of society in which he delights, but so it is, that the pains and penalties, from moral ills and physical maladies, make a sad drawback from the pleasures of life as drawn by our able author. Pleasure, he observes, is the "ordinary result of the action of the organs. Pain is sometimes the result, but it is the extraordinary, not the ordinary result." Abstracting, therefore, from the aggregate amount of pleasure, the aggregate amount of pain, "the balance in favour of pleasure is immense." 99. In respect to the amount of this balance, we own ourselves to be somewhat sceptical, and rather at variance with Dr. Smith. We hope and trust that the author's personal experience (for much of these calculations, after all, must be drawn from individual feelings) has prompted him to draw this flattering picture of human life. Happiness, he thinks, may exist without longevity—"but there cannot be longevity without happiness." There is, doubtless, much truth in this statement, unless an individual enjoy a tolerably good state of health, both mental and corporeal, long life is improbable.

"An advanced term of life and decrepitude are commonly conceived to be synonymous: the extension of life is vulgarly supposed to be the protraction of the period of infirmity and suffering, that period which is characterized by a progressive diminution of the power of sensation, and a consequent and proportionate loss of the power of enjoyment, the 'sans teeth, sans eyes, sans taste, sans every thing.' But this is so far from being true, that it is not within the compass of human power to protract in any sensible degree the period of old age properly so called, that is, the stage of decrepitude. In this stage of existence, the physical changes that successively take place clog, day by day, the vital machinery, until it can no longer play. In a space of time, fixed within narrow limits, the flame of life must then inevitably expire, for the processes that feed it fail. But though, when fully come, the term of old age cannot be extended, the coming of the term may be postponed. To the preceding stage, an indefinite number of years may be added. And this is a fact of the deepest interest to human nature." 112.

Dr. S. believes, and with reason, that the division of life into periods or epochs, is not an arbitrary distinction, but is founded on physiological differences in the constitution of man at certain stages of his existence. The first two years are ranked as infantile—the next six years as CHILDHOOD. At the age of eight years, he becomes a BOY—at sixteen years he is a "YOUNG MAN"—and at 24 years, he is an ADULT. In ten years more, viz. at the age of 34, "he will have acquired his highest state of physical perfection." From 34 to 48—that is, during a space of 14 years, man is considered by our author, as remaining nearly stationary. From 48, the meridian of mind and body, there is a gradual and regular decline of the intellectual and physical powers, till the age of decrepitude—84—after which the probability of life is about 4 years—the winter of old age, and the last scene of mortality!

We are not quite satisfied with the distribution of the epochs, and would rather divide them into septenniads, or periods of seven years, in each of which there is some moral or physical revolution of an interesting kind. If our author will compare page 113 with page 121, he will find a discrepancy in his own distribution. In the first mentioned page, infancy is circumscribed within *two* years;—in the other page, it is stated that "the period of infancy includes *precisely* THREE years." The errors, however, are very few, and the merits innumerable. We shall be exceedingly anxious for the appearance of the second volume, in which the *moral* attributes of man will be more particularly discussed, and in which the important subjects of education and hygiene will, we have no doubt, meet with particular attention from our author.

